# Gold Catalyzed Multicomponent Reactions beyond A^3^ Coupling

**DOI:** 10.3390/molecules23092255

**Published:** 2018-09-04

**Authors:** Renso Visbal, Sara Graus, Raquel P. Herrera, M. Concepción Gimeno

**Affiliations:** 1 Departamento de Ciencias Naturales y Exactas, Universidad de la Costa, Calle 58 #55-66, 080002 Barranquilla, Colombia; rvisbal4@cuc.edu.co; 2 Laboratorio de Organocatálisis Asimétrica, Departamento de Química Orgánica, Instituto de Síntesis Química y Catálisis Homogénea (ISQCH) CSIC-Universidad de Zaragoza, C/Pedro Cerbuna 12, E-50009 Zaragoza, Spain; sgraus@unizar.es; 3 Departamento de Química Inorgánica, Instituto de Síntesis Química y Catálisis Homogénea (ISQCH), CSIC-Universidad de Zaragoza, C/Pedro Cerbuna, No. 12, E-50009 Zaragoza, Spain

**Keywords:** β-alkoxyketones, butenolides, catalysis, ethers, gold, 3,4-dihydropyrimidin-2(1*H*)-ones, 1,4-dihydropyridines, multicomponent reactions, oxazoles, pyridines, spirocycles, thiazolo-quinolines

## Abstract

The preparation of complex architectures has inspired the search for new methods and new processes in organic synthesis. Multicomponent reactions have become an interesting approach to achieve such molecular diversity and complexity. This review intends to illustrate important gold-catalyzed examples for the past ten years leading to interesting skeletons involved in biologically active compounds.

## 1. Introduction

Multicomponent reactions (MCRs) have been defined as processes in which three or more reagents are added to a single vessel at the same time affording final products containing most of the atoms from the starting materials [1,2]. Therefore, more than one chemical transformation is involved in this approach without the necessity of changing the reaction media and without purification of the intermediates after each transformation. In the last years, MCRs have become powerful and efficient tools to afford molecular diversity, giving rise to libraries of small organic molecules while requiring less time and effort when compared with stop-and-go procedures [1,2]. These processes have also gained special interest in the pharmaceutical industry because of the easy formation of large libraries of compounds with potential biological activities [3].

This importance is reflected in the large number of publications reported in this field over the last decade [4,5]. Moreover, the utility and potential of multicomponent procedures have been confirmed by the development of a high number of molecules with remarkable biological activities [6,7,8,9,10].

Over the last two decades, gold catalysis has experienced a growing interest and an impressive development. Proof of that is the huge number of works published in this area of research [11,12,13]. The application of Au(I) in homogenous catalysis has been predominant [14,15,16,17], while reports based on Au(III) catalysis have been more scarce and have mainly focused on the application of simple salts such as AuCl_3_ or AuBr_3_ [18,19,20].

In relation with both fields of research, important contributions have been achieved with gold catalysts in the development of new multicomponent reactions, although many of these reactions have been only focused on the coupling of aldehydes, amines and alkynes known as A^3^-coupling-type reactions for the synthesis of propargylamines [21,22]. Since this process has already been compiled and revised in a previous pivotal review [23], we will give to the reader a broader vision of the scope of gold catalysts in the design and development of new MCRs beyond A^3^-coupling-type reactions.

**Figure 1:**
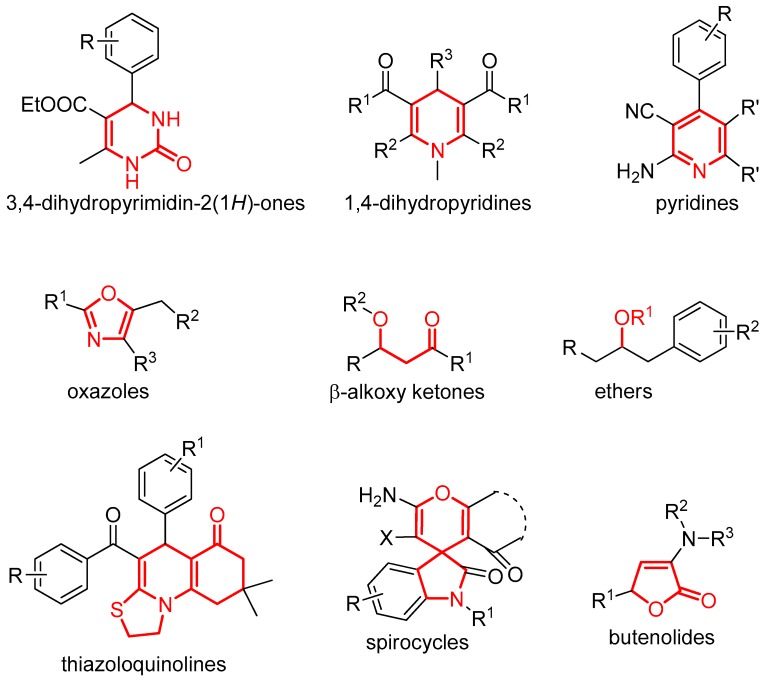
Structural cores described in this review and synthesized by means of gold-catalyzed MCRs.

In this context, we compile here some other representative pioneer multicomponent reactions for the construction of functionalized molecules such as those disclosed in Figure 1. Nevertheless, the field of multicomponent reactions using gold catalysts still needs a future progress and growth in comparison with other metals or approaches. Beyond the molecules depicted in Figure 1, other interesting protocols using gold catalysts have also been developed [24,25,26,27,28].

## 2. Gold-Catalyzed Multicomponent Reactions

### 2.1. Multicomponent Synthesis of 3,4-Dihydropyrimidin-2(1H)-ones. A Biginelli Approach

3,4-Dihydropyrimidin-2(1*H*)-ones **4** (DHPMs) have attracted considerable attention due to their antiviral, antitumor or anti-inflammatory activity, as well as their use as calcium channel blockers and as backbones of anticancer drugs [29,30,31]. The most common route to prepare DHPMs is the use of a classical Biginelli MCR [32,33,34].

**Scheme 1:**
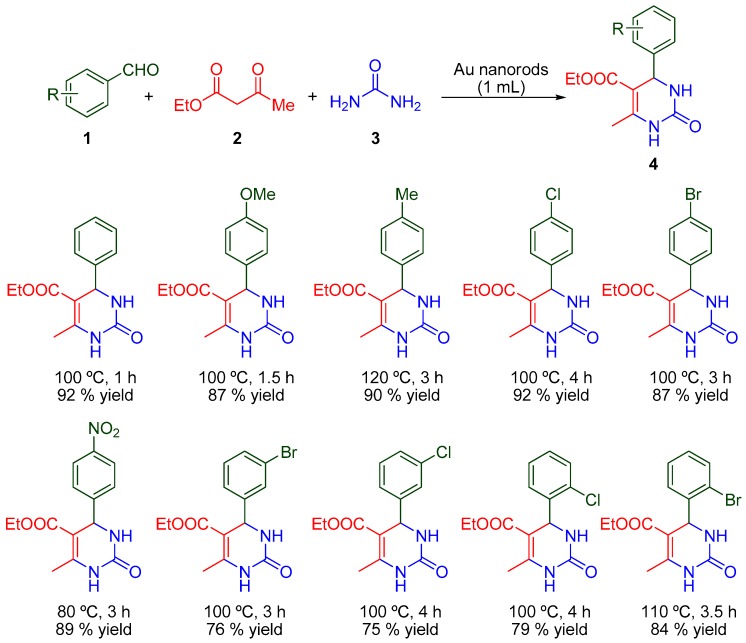
Biginelli reaction catalyzed by Au nanorods.

Tran and co-workers reported the first use of unsupported Au nanorods to catalyze the Biginelli reaction [35]. The authors prepared gold nanoparticles in different shapes, such as nanospheres, nanorods and nanostars and developed the Biginelli MCR with the gold nanorods. After a preliminary screening of the reaction conditions, the reaction was utilized to prepare a variety of aromatic aldehydes using 1.0 mL of Au nanorod colloid at 80–120 °C (Scheme 1) [35].

The final DHPMs were obtained in all cases with high yields, following a simple experimental procedure with a high catalyst efficiency. Moreover, the protocol allowed the elimination of volatile organic solvents and carrying out the reactions without an inert atmosphere Unfortunately, the authors did not propose a plausible reaction mechanism.

### 2.2. Multicomponent Synthesis of 1,4-Dihydropyridines (1,4-DHPs)

**Figure 2:**
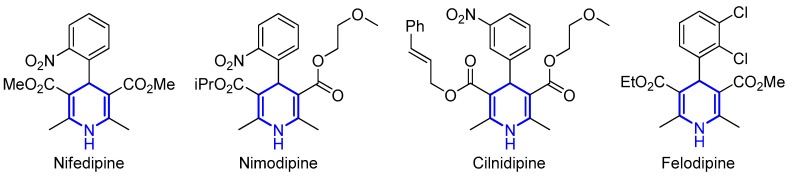
Biologically active 1,4-DHPs.

The 1,4-dihydropyridine (1,4-DHP) structural core, commonly synthesized following a classical Hantzsch condensation [36], has also attracted the attention of different research groups due to its appealing biological activity, including, among other properties, antioxidant, antidiabetic or antitumor effects (Figure 2) [37,38,39]. Therefore, their synthesis in racemic and chiral version is still an active area of research that includes multicomponent approaches [40,41,42,43,44].

**Scheme 2:**
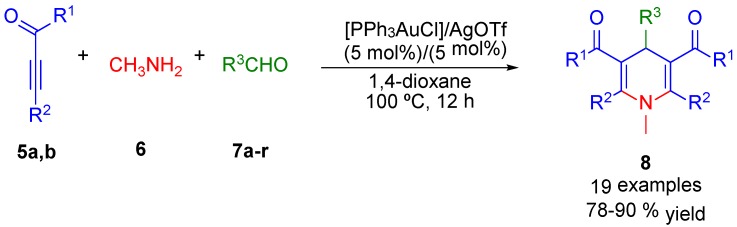
Au(I)-catalyzed multicomponent synthesis of 1,4-dihydropyridines **8**.

In this domain of research Cao and co-workers pioneered a gold-catalyzed multicomponent example to synthesize *N*-substituted DHPs **8**, through reaction of alkynes **5a**,**b**, methanamine **6** and different substituted aldehydes **7a**–**r** (Scheme 2). After an exploration of potential catalysts, base additives, solvent and temperature, the scope of the reaction was explored with the best reaction conditions (Scheme 2) [45].

**Scheme 3:**
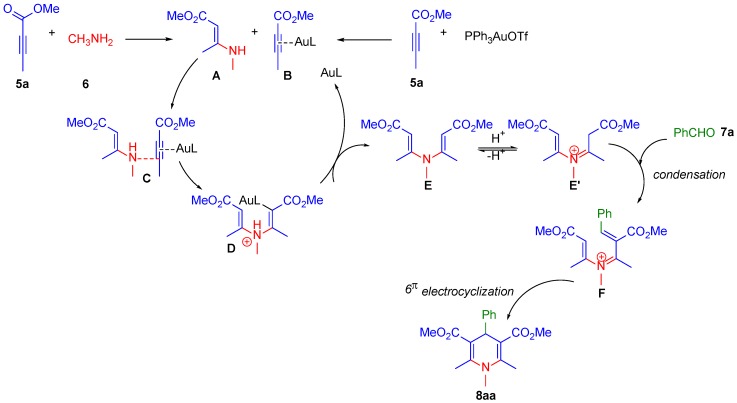
Proposed catalytic cycle for the synthesis of **8aa**.

This convergent approach, by generation of C–C and C–N bonds, affords *N*-substituted DHPs derivatives **8** in good yields. On the basis of the experimental results, a plausible mechanism was suggested by the authors to explain the synthesis of 1,4-DHPs **8** (Scheme 3).

First, adduct **A** would be obtained after nucleophilic addition of **5a** with **6**, while the activation of **5a** by PPh_3_AuOTf would afford Au(I)–alkyne complex **B**. The interaction of species **A** and **B** could produce intermediate **C**, which can undergo C–N bond formation to generate polarized intermediate **D** prior to the formation of intermediate **E**. This process is facilitated by the electrophilicity enhancement of the C-C bond promoted by the previous coordination of the gold complex to the species **5a**. The iminium ion intermediate **E′** can be obtained through an isomerization pathway from **E**, which can condense with aldehyde **7a** affording intermediate **F** by the attack from **E′** on the carbonyl carbon atom of the benzaldehyde. Finally, intermediate **F** can evolve to product **8aa** through a 6π electrocyclization followed by a deprotonation process.

### 2.3. Multicomponent Synthesis of Pyridines

**Figure 3:**
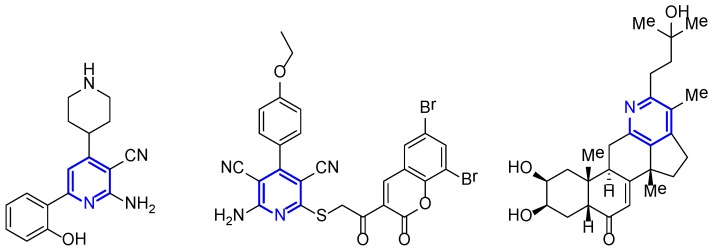
Biologically active pyridine derivatives.

Polysubstituted pyridines are compounds attracting great attention mostly because they constitute the skeleton of many natural products and organic functional materials, and also because they can be found in many synthetic compounds with pharmaceutical applications [46,47,48]. For example, 2-amino-3-cyanopyridine derivatives (Figure 3) have gained considerable attention due to their use as potent HIV-1 inhibitors [49,50].

**Scheme 4:**
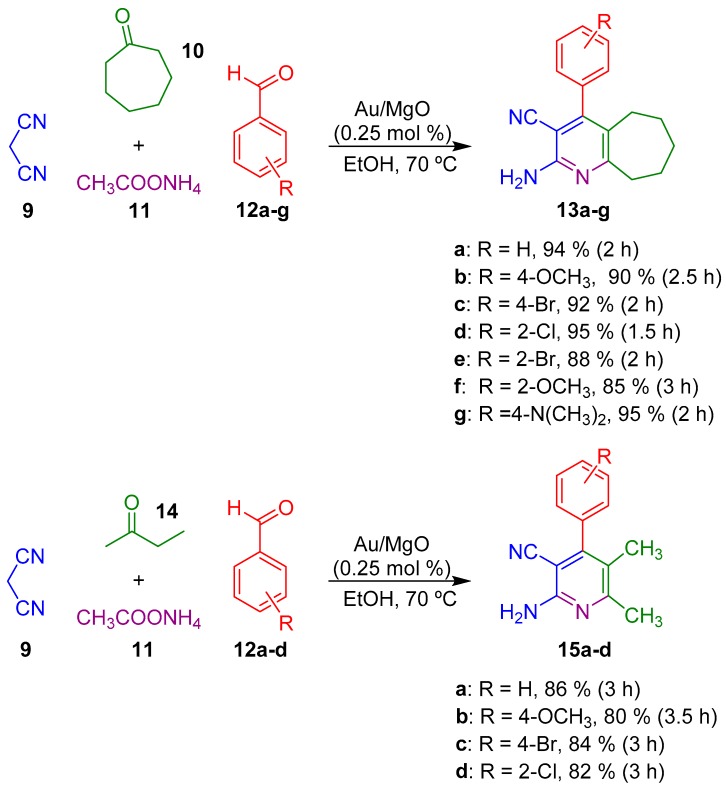
Multicomponent synthesis of highly substituted pyridines **13** and **15**.

In this field, Jonnalagadda and co-workers developed an efficient and reusable Au/MgO catalyst to promote the multicomponent synthesis of highly substituted pyridines **13** and **15** in high yields and in short reaction times (Scheme 4) [51].

**Scheme 5:**
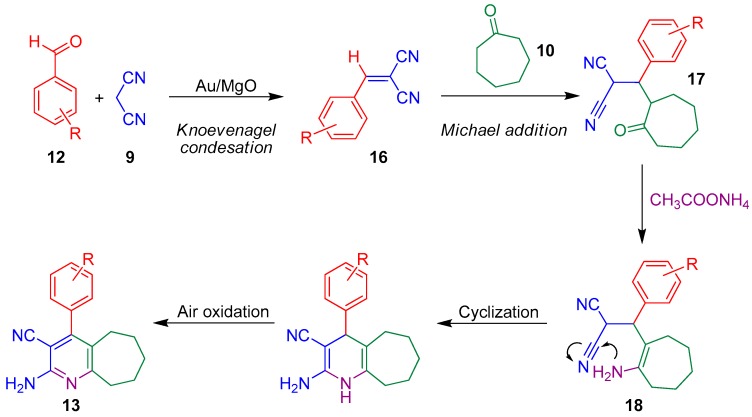
Proposed stepwise mechanism for the preparation of pyridines **13**.

A plausible reaction mechanism for the preparation of these highly substituted pyridines has been proposed by Jonnalagadda and co-workers. The first step consists on the formation of the arylidenemalononitrile intermediate **16** through a standard Knoevenagel condensation. The subsequent Michael-type addition of ketone **10** to the activated double bond of the arylidene affords species **17**. Finally, condensation with ammonia, followed by the cyclization of the resulting enamine **18** and the corresponding oxidation step forms compounds **13a**–**g** (Scheme 5) [51].

It should be noted that the catalyst was easily recovered and reused after the reaction without any loss of its catalytic activity during at least 5 runs.

### 2.4. Multicomponent Synthesis of Oxazoles

**Scheme 6:**
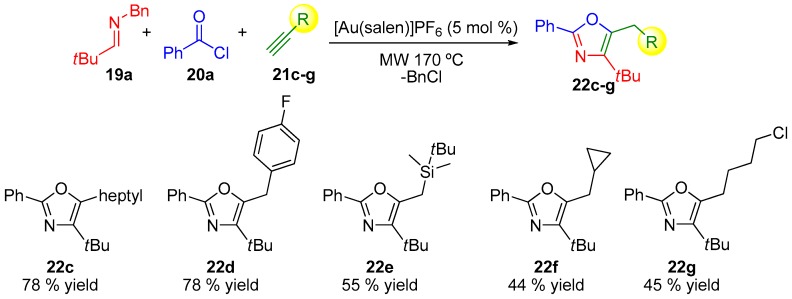
Structural variations in the alkyne compound **21** (salen: *N*,*N*′-ethylenebis(salicylimine)).

**Scheme 7:**
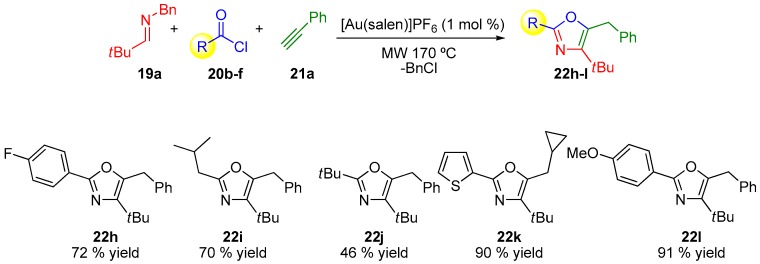
Structural variations in the acyl chlorides **20**.

**Scheme 8:**
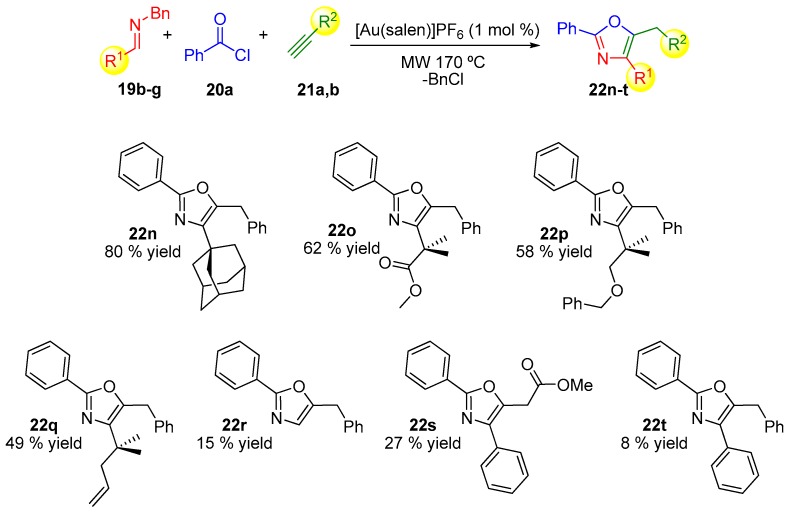
Structural variations in the imines **19**.

The oxazole motif is found in many compounds with useful applications and their synthesis is an active task in different areas of research [52,53,54,55,56,57,58,59,60]. In 2013, Strand and co-workers pioneered the first efficient gold-catalyzed three-component domino reaction to generate trisubstituted oxazoles directly from imines, alkynes, and acid chlorides (Scheme 6, Scheme 7 and Scheme 8) [61]. The use of MCRs had already been used for the synthesis of this family of products [62,63,64] and more recently Cai and co-workers have also pioneered a gold-catalyzed example [65]. The scope of the reaction was investigated by varying the corresponding alkyne compound **21** (Scheme 6), the acyl chloride **20** (Scheme 7) or the *N*-benzylimines **19** (Scheme 8), which provides variation on three substituted positions in the final oxazole ring [61].

**Scheme 9:**
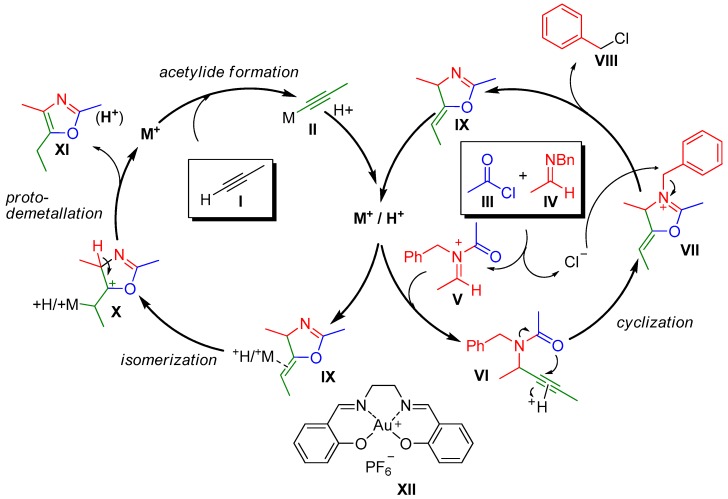
Proposed mechanism for the gold-catalyzed formation of oxazoles **XI**.

Several qualitative kinetic experiments confirmed that gold was not significantly involved in the cyclization step but did contribute to the isomeric transformation of **IX** into **X** (Scheme 9). Those observations led to propose the addition of metal acetylide **II** to the activated *N*-acyl iminium salt **V** to give propargyl amide as the initial step for the formation of the oxazole derivative. The acylation of amine (**IV**) induces the release of a chlorine anion that can subsequently react with **VII** to produce intermediate **IX** and the respective benzyl chloride (**VIII**) release. Finally, the isomerization of **IX** into the desired oxazole **XI** is achieved by a synergistic process between a Brønsted acid and a metal-mediated catalysis [66]. The same research group has also reported a more recent example of a gold-catalyzed multicomponent reaction for the synthesis of oxazoles [67].

### 2.5. Multicomponent Synthesis of β-Alkoxy Ketones

**Figure 4:**
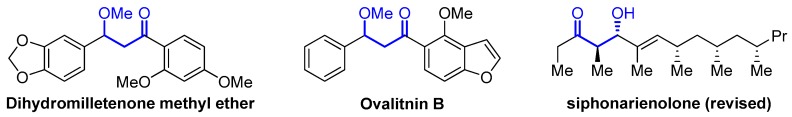
Biologically active β-alkoxy ketones.

β-Alkoxyketones are valuable building blocks in organic synthesis [68,69], and this structural core is present in many biologically active compounds and natural products [70,71,72], such as those disclosed in Figure 4. They are commonly synthesized either via aldol reaction or sequential epoxidation and reduction of enones.

**Scheme 10:**
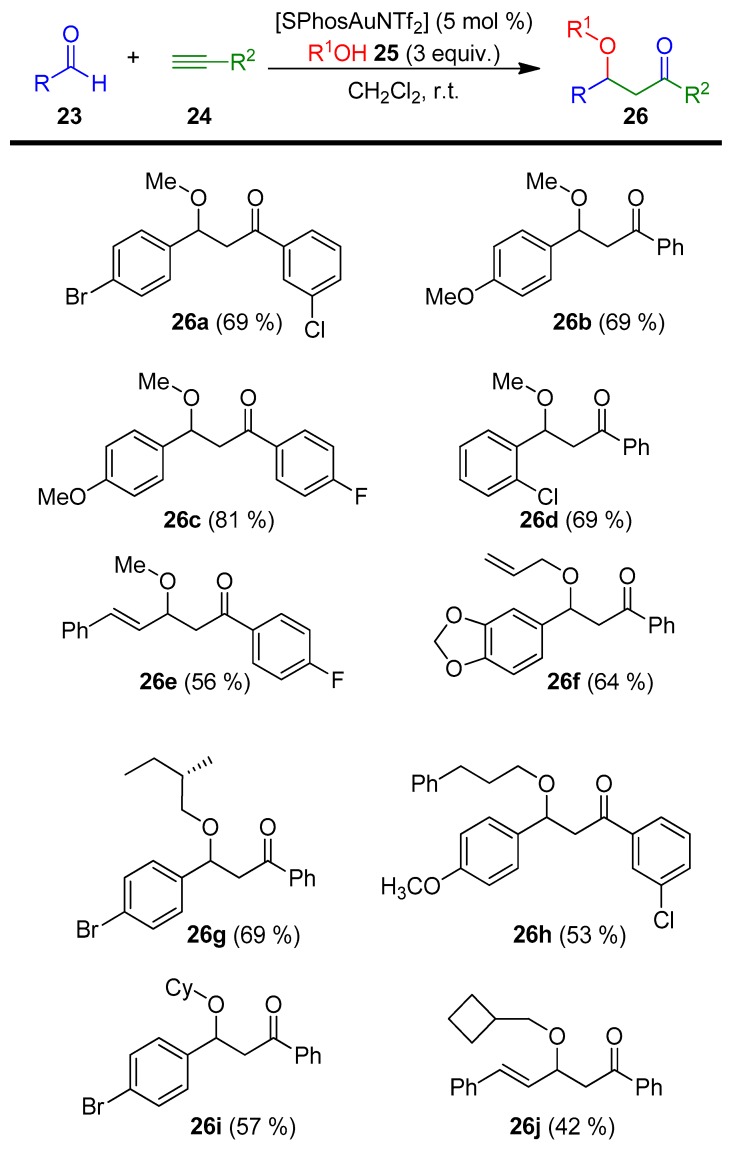
Gold(I)-catalyzed multicomponent synthesis of β-alkoxyketones **26**.

In this field, a gold(I)-catalyzed multicomponent synthesis of β-alkoxyketones from readily available precursors such as aldehydes **23**, alcohols **25**, and alkynes **24** was described in 2012 by Schultz and co-workers. The authors used the gold complex SPhosAuNTf_2_ as a catalyst to prepare diverse β-alkoxyketone products **26** (Scheme 10) [73].

With the best reaction conditions, the scope of this approach was explored by varying the aldehyde **23**, alkyne **24**, and alcohol **25** reaction partners. The resulting β-alkoxyketones **26** were obtained from moderate to good yields.

**Scheme 11:**
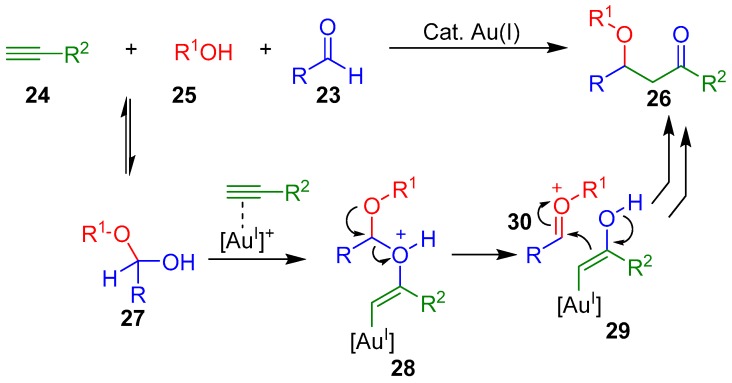
Previously proposed mechanism for Au(I)-catalyzed synthesis of β-alkoxyketones **26** via oxyauration of hemiacetals.

The authors proposed that an intermolecular oxyauration reaction between the alkyne **24** and the hemiacetal **27**, generated in situ by the reaction of the aldehyde with one equivalent of the alcohol **25**, could provide **28**, which could subsequent split into gold(I) enol **29** and oxocarbenium ion **30** (Scheme 11). Both intermediates could then undergo an intermolecular addition to furnish the β-alkoxyketone product **26** [74].

### 2.6. Multicomponent Oxidative Oxyarylation of Alkenes

Toste’s group reported the first example of a three-component gold-catalyzed oxidative oxyarylation of alkenes, in which alcohols, carboxylic acids, and even water were employed as nucleophiles in this pioneering protocol [75].

The desired ethers **33** were obtained in moderate to high yield when using several primary and secondary alcohols and even acids **31**, 5-phthalimidopentene **30a** and phenylboronic acid **32a** (Table 1).

With these results the authors also explored the variation of the arylboronic acid **32**, maintaining the alkenylamine **30a** and using MeOH as nucleophile. The final products **34** were obtained in good yields (Table 2).

The authors also checked the viability of this protocol using simple alkenes **35**, which were also successful as suitable substrates for alkoxy and acyloxyarylation under the optimized conditions (Table 3).

**Scheme 12:**
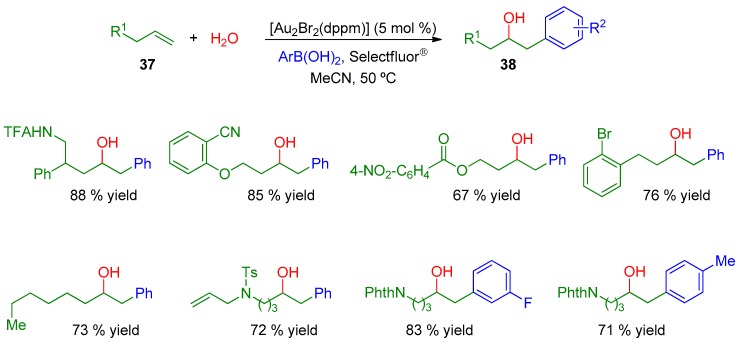
Use of water as nucleophile in the hydroxyarylation process (TFAHN: trifluoroacetamide; TsN: toluensulfonamide).

Interestingly, water was also employed as an appropriate nucleophile for the formation of alcohols **38** directly via hydroxyarylation, avoiding the deprotection of the previous alkoxy compounds (Scheme 12).

**Scheme 13:**
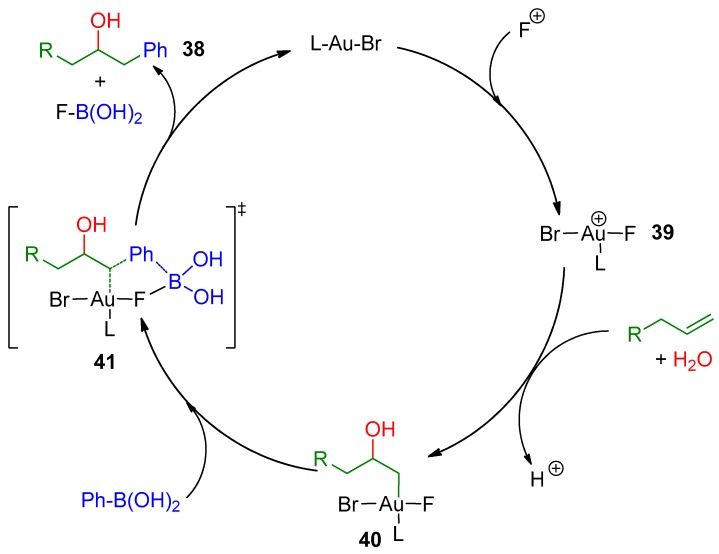
Plausible mechanism of the hydroxyarylation process.

A plausible mechanism for the formation of the alcohols **38** was proposed (Scheme 13). The mechanism is similar to one previously reported by the same authors for a related intramolecular aminoarylation reaction [76]. Hence, first, oxidation of the gold(I) bromide could occur generating a cationic gold(III) species **39**, which would activate the alkene toward the nucleophilic attack of H_2_O. Then, an oxyauration followed by C-C bond formation of **40** with the boronic acid could happen (**41**). Regeneration of the gold catalyst and release of the FB(OH)_2_ species would afford the final alcohol **38** (Scheme 13).

### 2.7. Multicomponent Synthesis of Thiazoloquinolines

**Scheme 14:**
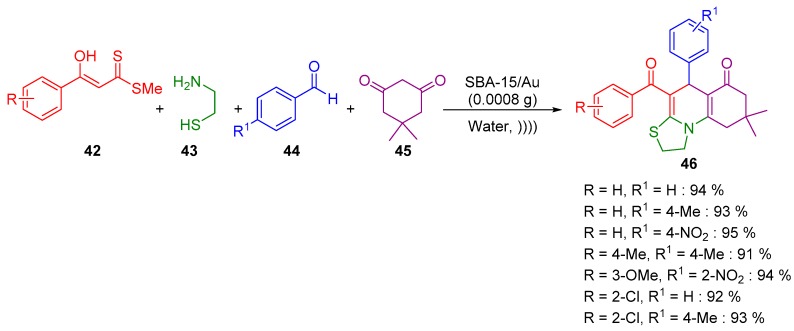
Synthesis of thiazoloquinolines **46**.

The use of microwave irradiation or sonication has also provided further possibilities to perform chemical reactions, tuning the selectivity in organic synthesis [77]. Sadeghzadeh developed a green approach for the four-component synthesis of thiazoloquinolines **46** using gold(III) dipyridine complex immobilized on SBA-15 as nano catalysts at room temperature (Scheme 14) [78], based on a previously reported work by Singh and co-workers [79].

After a broad exploration of the synthesis of gold nanoparticles and the optimization of the reaction conditions, the authors successfully developed an efficient synthesis of thiazoloquinolines **46** in very good yields using ultrasound.

It is worth noting that the gold catalyst could be efficiently recovered from the reaction mixtures. The activity of the recycled catalyst was also evaluated under optimized conditions of temperature, reaction time and the amount of catalyst. The gold catalyst was reused up to seven times. After each catalytic cycle, ICP technique was used to estimate whether the reaction took place at the surface of the SBA-15/Au catalyst. They found that the amount of Au(III) remaining after the seventh cycle was of about 2.1%. This value suggested good stability of the heterogeneous gold catalyst under the reaction conditions employed.

### 2.8. Multicomponent Synthesis of Spirocycles

**Figure 5:**
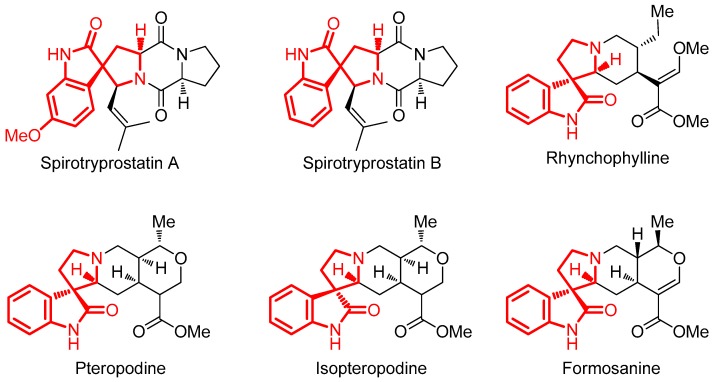
Spirotryprostatins A and B [89], pteropodine and isopteropodine [90], rhynchophylline [91] and formosanine [92].

A series of compounds containing an indole [80,81,82,83] or isatin [84,85,86] core have been described to possess important biological activities. Therefore, the effect of these structures in the generation of spirocycles could highly enhance biological activity of the resulting compounds [87]. Moreover, the spirooxindole ring is involved in many pharmaceuticals and natural products as shown in Figure 5 [88].

**Scheme 15:**
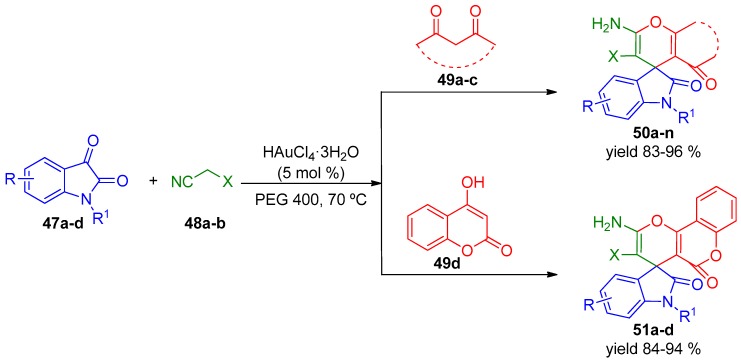
HAuCl_4_·3H_2_O catalyzed multicomponent synthesis of spirocycles **50** and **51**.

**Scheme 16:**
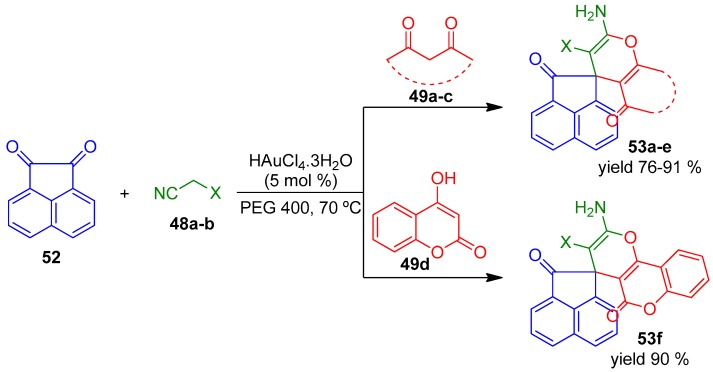
HAuCl_4_·3H_2_O catalyzed multicomponent synthesis of spirocycles **53**.

The Kidwai group developed a protocol for the efficient synthesis of functionalized spirochromenes **50** and **51** mediated by a gold(III) catalyst [93]. They used PEG 400 as it is an eco-friendly solvent medium, being inexpensive, thermally stable, recyclable and biodegradable (Scheme 15 and Scheme 16) [94]. Several parameters were explored in order to optimize the processes. Thus, the effect of catalyst loading, the presence of various Lewis acids as catalysts, a screening of solvents and the study of temperature was also analyzed. With the optimal reaction conditions, the methodology was firstly evaluated by using different isatins **47**, activated methylene compounds **48** and cyclic 1,3-diketones **49** for the synthesis of a series of tetrahydrospiro[chromene-4,3′-indoline] derivatives **50** and **51** (Scheme 14).

All compounds **50** and **51** were obtained with very good results (up to 96% yield). The authors extended the protocol to another reactant acenaphthoquinone **52**, instead of isatins **47**, under the same reaction conditions to prepare a variety of spiroacenaphthyleneone derivatives **53a**–**f** with excellent yields (Scheme 16).

**Scheme 17:**
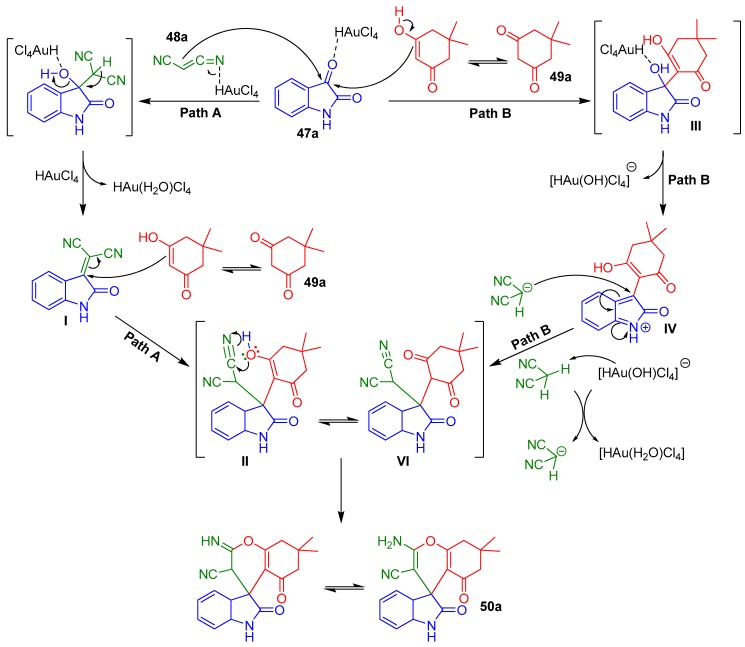
Plausible pathways for the synthesis of spirooxindole **50a**.

To explain the synthesis of spirooxindole derivatives **50** and **51** two plausible pathways were proposed based on previous studies [95] (Scheme 17).

Path A would start with a Knoevenagel condensation between isatin **47a** and malononitrile **48a** catalyzed by HAuCl_4_. This step would give rise to isatylidene malononitrile **I**. In a second step dimedone **49a** would attack to Knoevenagel adduct **I** via Michael addition producing intermediate **II** (path A). In contrast, path B would start with the attack of the dimedone **49a** to the isatin **47a** coordinated with HAuCl_4_ to afford the aldol adduct **III** followed by a subsequent dehydration of **III** and nucleophilic attack of **48a** to afford the intermediate **VI**. In both reaction pathways, the intermediate **II** would involve the cycloaddition between the hydroxyl group and the cyano moiety to form the desired spirooxindole **50a** (Scheme 17, path A and path B).

The recyclability of PEG 400 was also explored to proof the sustainability of the solvent. The solvent could be recycled with minimum loss and decomposition during three runs. The recycled PEG does not change in its reactivity but approximately 5% weight loss of PEG was observed after each completed cycle. However, the recyclability of the catalyst after the extraction of the product was accompanied by considerable leaching of the gold(III) chloride catalyst, avoiding its recyclability.

### 2.9. Multicomponent Synthesis of Butenolides

**Scheme 18:**
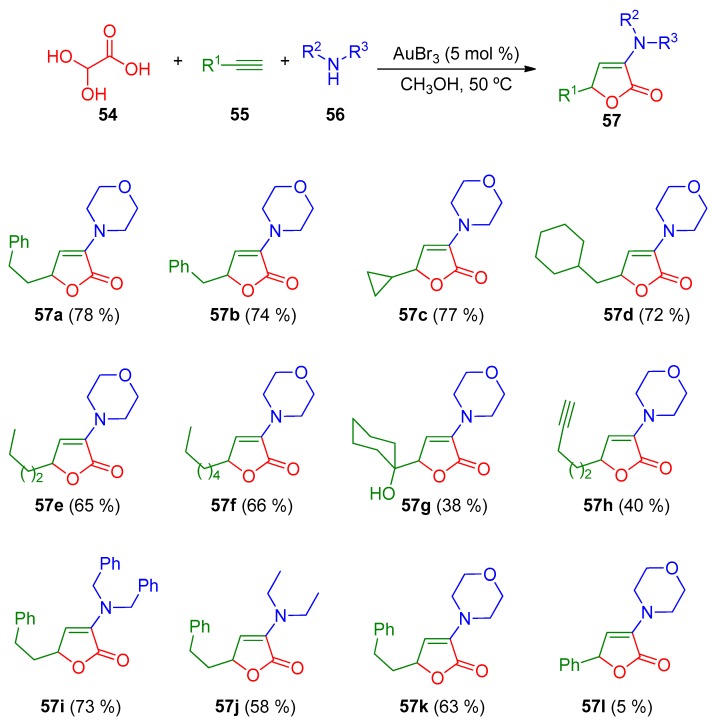
Gold-catalyzed multicomponent synthesis of butenolides **57**.

**Scheme 19:**
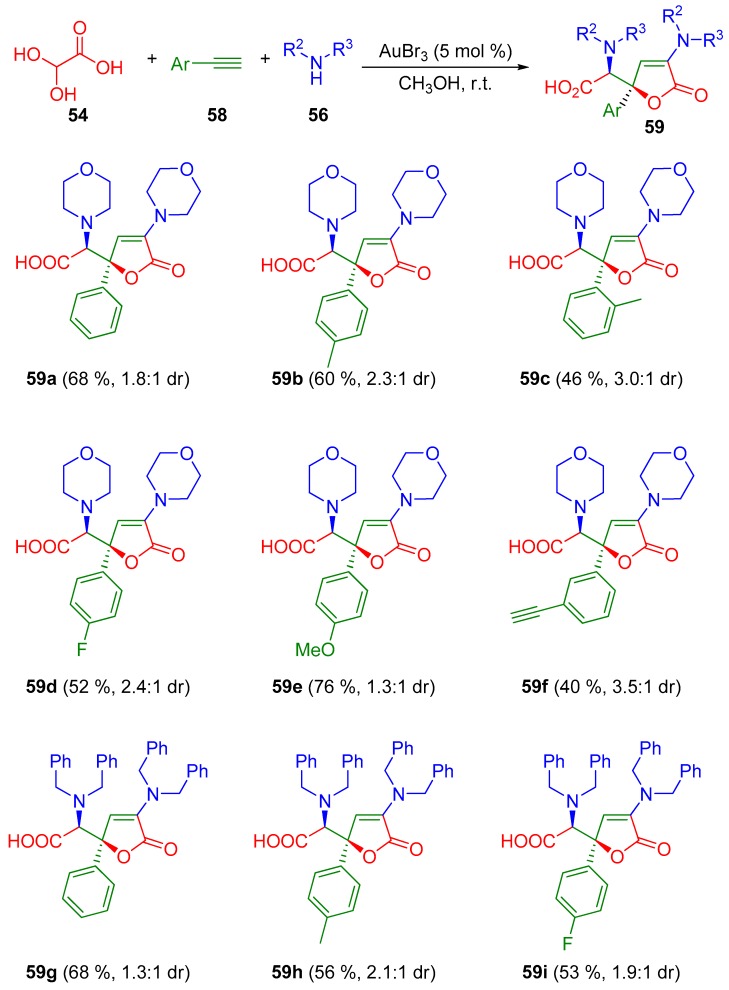
Gold-catalyzed tandem process for the synthesis of butenolides **59**.

The butenolide scaffold is also found in many natural products [96]. Moreover, they can exhibit interesting biological activities as antibiotic, antifungal, antifouling, or anticancer, among other properties [97,98,99,100]. In 2010, Ji and co-workers developed a gold-catalyzed three-component tandem approach between commercially available alkynes, amines and glyoxylic acid for the generation of two types of butenolides **57** and **59** (Scheme 18 and Scheme 19) [101].

**Scheme 20:**
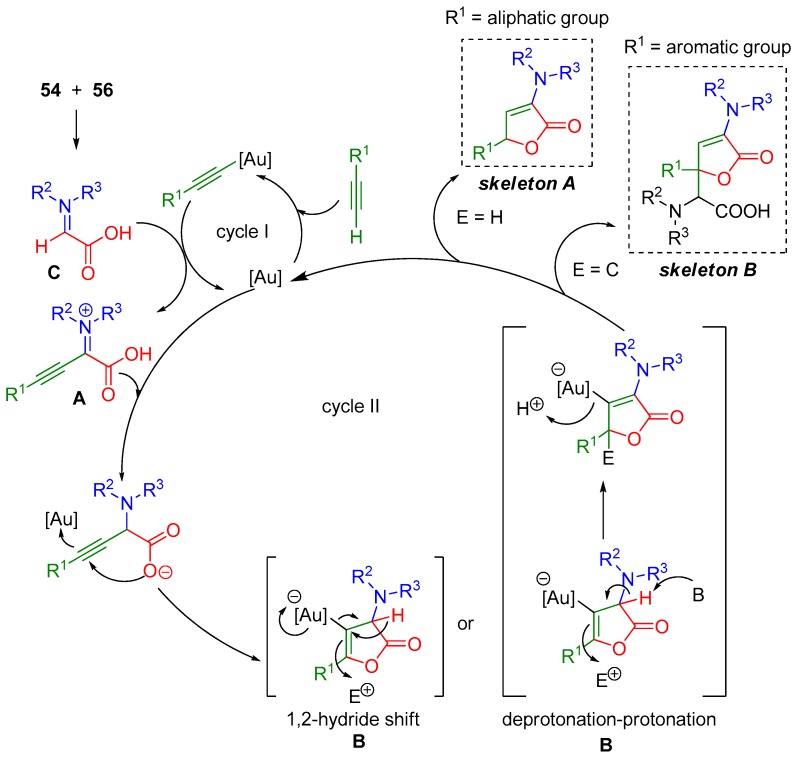
Catalytic cycle for the synthesis of butenolides **57** and **59**.

The authors proposed a plausible reaction mechanism based on their experimental results and previous studies (Scheme 20) [102,103,104]. The mechanism is suggested to be a tandem reaction involving two catalytic cycles (I and II). Firstly, in cycle I a gold-catalyzed three component coupling reaction between an alkyne, an amine, and glyoxylic acid occurs to give intermediate A. Next, gold-promoted endo-dig cyclization of A giving rise to intermediate B. Active intermediate B is subsequently transformed to butenolides **57** or **59** via electrophilic trapping along with a 1,2-hydride shift pathway or deprotonation-protonation sequence (Scheme 20). This gold-catalyzed multicomponent tandem protocol provides a valuable synthetic route to butenolides, expanding the area of gold catalysis.

## 3. Conclusions

The preparation of complex architectures has inspired the search for new methods and new processes in organic synthesis. In this sense, multicomponent reactions have become interesting protocols to achieve such molecular diversity and complexity. This objective, joined to the interest in the chemistry of gold, has allowed the synthesis of model structures using new efficient approaches. This review has illustrated these important and scarce examples in order to give a general vision related to the application and scope of gold catalysts in multicomponent reactions, until now dominated by the A^3^ coupling reaction. However, here it has been demonstrated that new processes have been developed, for which there is a preponderance of alkynes, carbonyls and amines as components for the coupling reactions. The plausible catalytic cycles to understand the role of gold catalyst in addition of each reagent involved have also been discussed in this work, and they clearly state the high Lewis acid capacity of gold catalysts to activate alkyne or carbonyl groups. The increasing interest in both gold catalysis and the development of more sustainable an economical procedures, make that a significant progress in the next few years is expected, with the use of gold catalysts for the discovery of new coupling reactions involving novel components that will allow the preparation of more complex scaffolds with potentially interesting properties.

## Figures and Tables

**Table 1:**
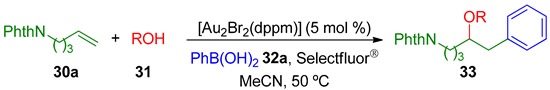
Alkoxy- and acyloxyarylation using different alcohols **31**.

Entry	R	Yield (%)	Entry	R	Yield (%)
1	Me	79	7	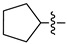	85
2	Et	85	8	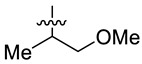	88 d.r. 1:1
3	*i*Pr	90	9	Me(CO)-	62
4	(Me)_3_CCH_2_	91	10	Et(CO)-	69
5	*t*Bu	33	11	*i*Pr(CO)-	50
6	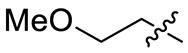	85	12	Ph(CO)-	48

Dppm: diphenylphosphinomethane; PhthN: phthalimido.

**Table 2:**
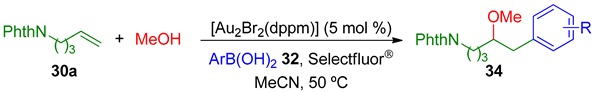
Methoxyarylation with different ArB(OH)_2_ **32**.

Entry	R	Yield (%)	Entry	R	Yield (%)
1	4-Me	88	7	4-MeO_2_C	83
2	4-Br	90	8	4-H(CO)	82
3	3-F	79	9	3,5-bis-CF_3_	77

**Table 3:**
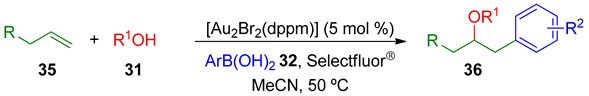
Alkoxy- and acyloxyarylation of simple alkenes **35**.

Entry			R^1^	R^2^	Yield (%)
1	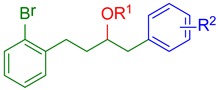		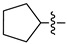	4-Br	69
2			Me	2-CO_2_Me	78
3			Me(CO)	4-Br	51
4	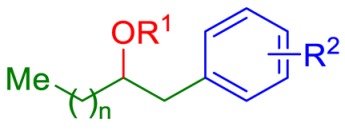	n = 5	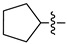	4-Br	76
5		n = 5	(Me)_3_CCH_2_	4-Br	73
6		n = 9	(Me)_3_CCH_2_	H	66
7		n = 5	Me(CO)	4-Br	53
